# Supplementary material for: A Likelihood Approach for Real-Time Calibration of Stochastic Compartmental Epidemic Models
Source: PLoS Comput Biol. 2017 Jan 17;13(1):e1005257. doi: 10.1371/journal.pcbi.1005257 (PMC5240920; doi:10.1371/journal.pcbi.1005257)
Supplement: S1 File — (TAR.GZ) [file pcbi.1005257.s014.tar.gz › HSPH_Online-SI-Revision/PF08/n10-mild/n10-mild_PF_table-4.pdf]

1	R0	{1.25046, 6.80278}	{1.5491, 19.7081}	{{1.51737, 19.1766}, {1.58827, 20.}}	{23.8826, 189.706}	inf	157.	685.705	{618.317, 726.252}	336.754	0.639071	newly	177.	249.61	{221., 281.}	41.0226	0.147443	Reff	1.16118	1.21558	{1.18766, 1.23873}	4.68529	ar	3516.	7157.45	{6885., 7413.}	103.568
2	R0	{1.32013, 4.45847}	{1.696, 18.6778}	{{1.47583, 14.3616}, {2.0337, 20.}}	{28.4724, 318.929}	inf	184.	601.251	{389.881, 807.74}	226.767	0.498129	newly	307.	243.19	{162., 331.}	25.7492	0.135139	Reff	1.21267	1.45564	{1.27252, 1.5788}	20.05	ar	4420.	7531.03	{6488., 8390.}	70.3853
3	R0	{1.31785, 5.36796}	{1.73924, 16.647}	{{1.36826, 11.9534}, {2.25421, 20.}}	{32.0074, 210.119}	inf	142.	486.572	{241.619, 813.213}	242.656	0.487176	newly	232.	227.29	{113., 371.}	36.8836	0.169801	Reff	1.23206	1.5409	{1.2609, 1.92639}	25.2113	ar	4942.	7241.16	{5685., 9001.}	46.8588
4	R0	{1.38801, 6.16031}	{1.58337, 19.4706}	{{1.47645, 18.8167}, {1.70627, 20.}}	{14.0749, 216.066}	inf	202.	647.272	{518.337, 821.196}	220.432	0.498865	newly	273.	246.26	{191., 311.}	16.9451	0.0804284	Reff	1.26267	1.34509	{1.28471, 1.42748}	7.06073	ar	4790.	7377.05	{6852., 8006.}	54.0094
5	R0	{1.27955, 11.57}	{1.52023, 19.5342}	{{1.5115, 19.4287}, {1.53114, 19.5927}}	{18.8098, 68.8346}	inf	185.	362.939	{357.933, 367.248}	96.1832	0.292616	newly	121.	121.59	{108., 133.}	6.68595	0.0291211	Reff	1.13842	0.692711	{0.685955, 0.698575}	39.1513	ar	3460.	7156.87	{7047., 7264.}	106.846
6	R0	{1.22184, 4.34395}	{1.77312, 17.0355}	{{1.39701, 12.2751}, {2.16609, 20.}}	{45.1189, 292.167}	inf	108.	362.015	{189.521, 584.403}	235.199	0.486446	newly	215.	174.62	{83., 295.}	37.0977	0.203782	Reff	1.17138	1.63087	{1.30032, 1.96827}	39.2265	ar	3524.	7383.25	{5708., 8861.}	109.513
7	R0	{1.23856, 4.59068}	{1.6172, 18.7093}	{{1.42015, 13.339}, {1.82762, 20.}}	{30.5711, 307.55}	inf	160.	577.433	{395.165, 823.803}	260.895	0.540626	newly	265.	230.59	{160., 315.}	22.8792	0.113453	Reff	1.13155	1.396	{1.24811, 1.54258}	23.3712	ar	3855.	7189.63	{6175., 8274.}	86.5014
8	R0	{1.34531, 10.6689}	{1.56158, 19.8134}	{{1.51877, 19.3414}, {1.61499, 20.}}	{16.0757, 85.7121}	inf	312.	741.188	{674.197, 825.47}	137.56	0.374479	newly	198.	269.51	{242., 297.}	36.1162	0.132352	Reff	1.20365	1.21665	{1.19299, 1.23636}	1.44923	ar	4852.	7338.76	{7056., 7635.}	51.2523
9	R0	{1.31775, 5.19226}	{1.63924, 19.3063}	{{1.51033, 17.9397}, {1.84257, 20.}}	{24.3972, 271.829}	inf	120.	623.883	{459.411, 853.053}	419.902	0.705148	newly	202.	243.69	{179., 334.}	26.0842	0.0957834	Reff	1.22551	1.40653	{1.32052, 1.519}	14.8118	ar	4708.	7562.15	{6820., 8409.}	60.6234
10	R0	{1.25916, 6.89207}	{1.57095, 19.6468}	{{1.51878, 18.993}, {1.62388, 20.}}	{24.7615, 185.064}	inf	175.	744.343	{647.155, 876.122}	325.339	0.625661	newly	191.	276.96	{232., 329.}	45.0052	0.157849	Reff	1.10151	1.25436	{1.22245, 1.28631}	13.9789	ar	3823.	7513.17	{7194., 7870.}	96.5255
11	R0	{1.20377, 5.46566}	{1.64381, 18.068}	{{1.44226, 13.1706}, {1.87803, 20.}}	{36.555, 230.574}	inf	147.	500.364	{344.463, 724.518}	240.384	0.5161	newly	200.	213.	{144., 290.}	24.12	0.0992021	Reff	1.11577	1.45765	{1.28306, 1.66056}	30.6404	ar	3114.	7320.43	{6222., 8395.}	135.081
12	R0	{1.27878, 2.55804}	{1.65327, 17.6956}	{{1.3513, 12.1904}, {2.03884, 20.}}	{29.2846, 591.764}	inf	57.	319.708	{195.418, 577.791}	460.892	0.718153	newly	277.	140.22	{84., 237.}	50.0072	0.32902	Reff	1.24259	1.52142	{1.26411, 1.79299}	22.8265	ar	3930.	6909.51	{5309., 8549.}	75.8145
13	R0	{1.41227, 5.45696}	{1.55403, 19.6918}	{{1.50334, 19.0879}, {1.59703, 20.}}	{10.0378, 260.856}	inf	188.	708.012	{635.088, 787.593}	276.602	0.574303	newly	295.	257.5	{226., 294.}	13.7559	0.0654904	Reff	1.28587	1.21465	{1.19124, 1.23918}	5.53859	ar	4822.	7243.95	{6945., 7553.}	50.2271
14	R0	{1.32917, 7.27593}	{1.58168, 19.6805}	{{1.50759, 18.9787}, {1.66101, 20.}}	{18.9977, 170.487}	inf	250.	746.269	{665.645, 868.853}	198.508	0.472182	newly	248.	274.74	{240., 330.}	12.629	0.0497482	Reff	1.18482	1.27762	{1.24223, 1.31812}	7.87265	ar	4758.	7582.55	{7268., 7980.}	59.3642
15	R0	{1.31629, 9.97481}	{1.57218, 19.4293}	{{1.52513, 18.6832}, {1.6482, 20.}}	{19.4403, 94.7836}	inf	186.	592.368	{481.382, 750.262}	218.478	0.497065	newly	149.	224.75	{182., 291.}	50.8389	0.172052	Reff	1.22178	1.33357	{1.29736, 1.3676}	9.15013	ar	4543.	7213.79	{6797., 7679.}	58.7891
16	R0	{1.36735, 5.74166}	{1.58463, 18.8337}	{{1.42229, 17.3006}, {1.79199, 20.}}	{15.9208, 228.018}	inf	182.	534.027	{353.826, 777.625}	193.421	0.451144	newly	266.	210.44	{140., 292.}	26.3835	0.139136	Reff	1.26507	1.36852	{1.22161, 1.50926}	9.63218	ar	4516.	7017.57	{6134., 8030.}	55.3935
17	R0	{1.27524, 13.2396}	{1.55007, 19.7441}	{{1.51119, 19.0802}, {1.5859, 20.}}	{21.5513, 49.1291}	inf	80.	631.955	{583.907, 692.42}	689.944	0.896342	newly	45.	232.77	{207., 271.}	417.267	0.711476	Reff	1.24591	1.26728	{1.24665, 1.2862}	1.85415	ar	3731.	7212.23	{7021., 7454.}	93.3055
18	R0	{1.21884, 7.71545}	{1.55978, 19.7304}	{{1.50915, 19.2391}, {1.6081, 20.}}	{27.9726, 155.726}	inf	167.	674.491	{594.658, 780.893}	303.887	0.603908	newly	159.	249.55	{214., 297.}	56.9497	0.192572	Reff	1.1234	1.27245	{1.24594, 1.29258}	13.2675	ar	3080.	7326.	{6991., 7681.}	137.857
19	R0	{1.34879, 6.71464}	{1.70504, 18.0683}	{{1.46651, 13.076}, {2.05047, 20.}}	{26.4126, 169.088}	inf	339.	748.78	{487.641, 1087.61}	120.879	0.325587	newly	410.	309.29	{206., 427.}	27.0463	0.146954	Reff	1.15092	1.36831	{1.19795, 1.52151}	18.9377	ar	4585.	7526.8	{6399., 8665.}	64.1614
20	R0	{1.37284, 5.75036}	{1.70525, 17.8867}	{{1.42417, 12.9395}, {2.04795, 20.}}	{24.2552, 211.053}	inf	216.	640.231	{380.606, 849.541}	196.403	0.453683	newly	303.	270.8	{184., 349.}	18.5149	0.0927115	Reff	1.23597	1.43954	{1.23089, 1.64349}	17.013	ar	4750.	7550.36	{6085., 8739.}	58.9549
21	R0	{1.29938, 5.36419}	{1.69748, 18.2547}	{{1.44539, 13.8356}, {2.05369, 20.}}	{30.6376, 240.307}	inf	198.	634.119	{433.664, 883.354}	220.262	0.490689	newly	270.	264.89	{186., 365.}	21.3296	0.0970191	Reff	1.18387	1.44076	{1.251, 1.65073}	21.7351	ar	4337.	7495.05	{6277., 8731.}	72.8165
22	R0	{1.31882, 7.47626}	{1.55825, 19.7111}	{{1.49718, 19.3258}, {1.61659, 20.}}	{18.1547, 163.649}	inf	227.	688.753	{599.769, 804.689}	203.415	0.479416	newly	235.	256.84	{220., 296.}	12.017	0.0479175	Reff	1.193	1.26096	{1.22765, 1.29541}	5.69587	ar	3977.	7315.76	{6950., 7682.}	83.9517
23	R0	{1.26215, 3.46375}	{1.80241, 18.9991}	{{1.5051, 14.767}, {2.08349, 20.}}	{42.8044, 448.514}	inf	117.	587.438	{386.019, 810.607}	402.084	0.681684	newly	283.	240.01	{146., 331.}	25.424	0.133323	Reff	1.18314	1.57524	{1.35726, 1.75277}	33.1405	ar	3607.	7902.49	{6619., 8846.}	119.088
24	R0	{1.16712, 4.19464}	{1.58811, 19.235}	{{1.48982, 18.5381}, {1.67432, 20.}}	{36.0705, 358.562}	inf	122.	721.885	{577.888, 872.456}	491.709	0.76561	newly	227.	274.1	{218., 342.}	22.6608	0.0833406	Reff	1.04609	1.29196	{1.24505, 1.35328}	23.5036	ar	3000.	7388.85	{6931., 7909.}	146.295
25	R0	{1.22751, 3.34517}	{1.67598, 17.1501}	{{1.35302, 11.6846}, {2.08402, 20.}}	{36.535, 412.682}	inf	50.	301.695	{170.025, 460.89}	503.39	0.749763	newly	113.	140.01	{73., 201.}	41.5487	0.153551	Reff	1.20861	1.56377	{1.2744, 1.85666}	29.5224	ar	3086.	6962.44	{5204., 8585.}	125.614
26	R0	{1.18747, 5.88083}	{1.55934, 19.8192}	{{1.50687, 19.1862}, {1.63046, 20.}}	{31.3158, 237.014}	inf	178.	803.528	{722.235, 880.861}	351.42	0.652949	newly	234.	296.5	{264., 327.}	26.7094	0.101022	Reff	1.02301	1.22933	{1.20393, 1.26168}	20.169	ar	3614.	7528.81	{7194., 7839.}	108.323
27	R0	{1.29023, 4.76202}	{1.56533, 19.4417}	{{1.49463, 18.9091}, {1.6526, 20.}}	{21.3217, 308.266}	inf	206.	674.25	{576.551, 850.472}	227.306	0.510148	newly	330.	253.62	{211., 316.}	23.8	0.122209	Reff	1.17217	1.2954	{1.26152, 1.33854}	10.6039	ar	4245.	7274.75	{6841., 7813.}	71.3722
28	R0	{1.2258, 12.9056}	{1.51538, 19.9864}	{{1.50751, 20.}, {1.51705, 20.}}	{23.624, 54.8657}	inf	188.	236.393	{227.76, 237.874}	25.7411	0.0986053	newly	113.	77.79	{64., 90.}	31.1593	0.165576	Reff	1.0369	0.645776	{0.644232, 0.648348}	37.7207	ar	3639.	7727.67	{7619., 7832.}	112.357
29	R0	{1.34702, 6.29049}	{1.63483, 18.893}	{{1.44999, 16.1209}, {1.82873, 20.}}	{21.3662, 200.343}	inf	264.	699.734	{466.823, 1012.38}	165.051	0.40819	newly	312.	272.53	{195., 380.}	22.0737	0.108807	Reff	1.19265	1.32003	{1.20349, 1.43552}	11.1193	ar	4560.	7214.36	{6294., 8164.}	58.2096
30	R0	{1.37931, 6.78741}	{1.5897, 18.5377}	{{1.42963, 14.0088}, {1.79206, 20.}}	{15.2754, 173.119}	inf	251.	695.821	{483.201, 907.568}	177.219	0.430145	newly	287.	274.57	{196., 351.}	17.7317	0.0822985	Reff	1.17614	1.27873	{1.15127, 1.38929}	9.59677	ar	4667.	7106.25	{6272., 7956.}	52.2659
31	R0	{1.16062, 4.47529}	{1.72576, 17.1139}	{{1.38469, 11.8821}, {2.17791, 20.}}	{48.6931, 282.409}	inf	80.	336.523	{189.324, 513.397}	320.654	0.590149	newly	156.	159.41	{82., 250.}	34.8013	0.152143	Reff	1.12661	1.59413	{1.29463, 1.9718}	41.4975	ar	3037.	7236.26	{5597., 8849.}	138.27
32	R0	{1.37647, 18.7122}	{1.52902, 19.921}	{{1.49547, 19.6648}, {1.56319, 20.}}	{11.0827, 6.45998}	inf	360.	348.094	{326.186, 376.167}	5.5708	0.0250171	newly	137.	115.7	{100., 129.}	15.6204	0.0756443	Reff	1.09897	0.659178	{0.647441, 0.671614}	40.0188	ar	4824.	7559.56	{7392., 7726.}	56.7073
33	R0	{1.35084, 6.08841}	{1.61638, 19.4692}	{{1.51585, 18.9571}, {1.71943, 20.}}	{19.6571, 219.775}	inf	217.	660.33	{523.635, 796.24}	204.3	0.47744	newly	297.	252.17	{203., 310.}	17.5118	0.0872172	Reff	1.23859	1.37886	{1.31621, 1.45674}	11.3502	ar	4759.	7537.44	{7017., 8066.}	58.3829
34	R0	{1.36912, 6.316}	{1.60575, 19.3565}	{{1.49791, 18.5715}, {1.73355, 20.}}	{17.2836, 206.467}	inf	198.	660.096	{501.287, 824.223}	233.382	0.515134	newly	262.	253.31	{196., 314.}	16.355	0.0733665	Reff	1.2459	1.35048	{1.2865, 1.44717}	8.58527	ar	4537.	7404.19	{6834., 8092.}	63.1957
35	R0	{1.35831, 7.09038}	{1.67008, 18.301}	{{1.49022, 13.2931}, {1.89279, 20.}}	{22.9528, 158.11}	inf	331.	784.905	{564.286, 1142.6}	137.132	0.360234	newly	339.	320.15	{215., 430.}	18.8112	0.0878718	Reff	1.17304	1.3426	{1.21736, 1.49116}	14.5185	ar	4667.	7753.38	{6882., 8662.}	66.132
36	R0	{1.28315, 5.89938}	{1.69575, 18.741}	{{1.45348, 15.0627}, {2.01193, 20.}}	{32.1552, 217.678}	inf	255.	626.809	{414.173, 869.124}	145.807	0.375914	newly	336.	252.16	{170., 340.}	27.6548	0.149737	Reff	1.15291	1.43603	{1.24023, 1.62385}	24.5569	ar	4460.	7518.24	{6361., 8578.}	68.5704
37	R0	{1.19303, 2.75113}	{1.71706, 17.2314}	{{1.38298, 12.9845}, {2.14013, 20.}}	{43.9244, 526.34}	inf	55.	418.735	{214.429, 749.95}	661.336	0.83901	newly	184.	189.87	{102., 309.}	36.2337	0.154301	Reff	1.13565	1.55398	{1.27995, 1.88637}	36.837	ar	3142.	7209.84	{5631., 8809.}	129.467
38	R0	{1.36033, 7.8088}	{1.66644, 19.2667}	{{1.49541, 17.8812}, {1.87229, 20.}}	{22.5028, 146.73}	inf	249.	669.496	{475.377, 919.515}	168.874	0.419283	newly	256.	261.18	{185., 355.}	18.7188	0.0804824	Reff	1.22334	1.42058	{1.31154, 1.56973}	16.1972	ar	4941.	7780.2	{7051., 8659.}	57.4621
39	R0	{1.27353, 3.70845}	{1.63559, 17.7353}	{{1.42821, 13.3449}, {1.86599, 20.}}	{28.43, 378.24}	inf	114.	530.606	{337.27, 799.258}	365.444	0.646523	newly	257.	227.	{143., 329.}	23.1051	0.116819	Reff	1.17827	1.42618	{1.26453, 1.62041}	21.0403	ar	3725.	7241.97	{6029., 8364.}	94.4153
40	R0	{1.19983, 12.1486}	{1.48709, 19.6126}	{{1.46996, 19.5113}, {1.50583, 19.664}}	{23.9415, 61.4395}	inf	191.	198.259	{195.364, 201.521}	3.88228	0.0165052	newly	121.	66.47	{57., 76.}	45.0661	0.262503	Reff	1.02525	0.638638	{0.621319, 0.643344}	37.7094	ar	3619.	7277.59	{7165., 7423.}	101.094
41	R0	{1.20935, 2.67169}	{1.73746, 17.417}	{{1.38635, 13.7468}, {2.15093, 20.}}	{43.6693, 551.911}	inf	53.	360.761	{205.828, 597.284}	580.681	0.793124	newly	165.	167.93	{86., 302.}	37.8364	0.160844	Reff	1.16702	1.6011	{1.29804, 1.98055}	37.2136	ar	3429.	7232.91	{5579., 8878.}	110.934
42	R0	{1.23313, 4.74781}	{1.52708, 19.6777}	{{1.46089, 19.0494}, {1.59534, 20.}}	{23.838, 314.458}	inf	138.	663.92	{581.736, 767.643}	381.102	0.678895	newly	211.	243.72	{203., 284.}	17.5355	0.0678555	Reff	1.10217	1.24294	{1.21086, 1.27698}	12.772	ar	3835.	7097.15	{6766., 7464.}	85.0626
43	R0	{1.26105, 2.39713}	{1.67647, 17.9901}	{{1.40212, 13.8698}, {1.99404, 20.}}	{32.9421, 650.487}	inf	88.	398.731	{264.6, 538.104}	353.103	0.637801	newly	321.	173.3	{117., 230.}	46.4361	0.288568	Reff	1.20809	1.53697	{1.34823, 1.79096}	27.2232	ar	3723.	7399.37	{6107., 8736.}	98.7475
44	R0	{1.36948, 8.88006}	{1.59949, 19.5122}	{{1.50889, 18.6441}, {1.72229, 20.}}	{16.7952, 119.731}	inf	274.	645.983	{529.618, 801.614}	135.76	0.366483	newly	232.	245.45	{197., 300.}	15.056	0.0614105	Reff	1.23623	1.35417	{1.29524, 1.43278}	9.62037	ar	4437.	7413.05	{6881., 8038.}	67.0735
45	R0	{1.34882, 3.3791}	{1.82802, 16.9484}	{{1.37811, 11.0972}, {2.38071, 20.}}	{35.8498, 401.566}	inf	49.	304.801	{164.464, 512.339}	522.043	0.751035	newly	143.	149.74	{69., 255.}	40.8392	0.177923	Reff	1.32279	1.72355	{1.31293, 2.16844}	31.268	ar	4422.	7510.11	{5605., 9108.}	69.8808
46	R0	{1.23774, 4.34917}	{1.7066, 18.3114}	{{1.42469, 14.5534}, {2.03846, 20.}}	{37.8805, 321.032}	inf	107.	497.065	{321.94, 719.036}	364.547	0.64676	newly	199.	210.74	{130., 282.}	25.8191	0.105323	Reff	1.16533	1.53741	{1.3046, 1.79972}	31.9286	ar	3685.	7574.4	{6301., 8762.}	105.547
47	R0	{1.34327, 4.78811}	{1.66471, 18.2229}	{{1.44684, 13.0737}, {2.00958, 20.}}	{23.9297, 280.586}	inf	102.	396.173	{281.115, 571.821}	288.405	0.57479	newly	151.	167.74	{109., 240.}	26.2781	0.103813	Reff	1.29693	1.52451	{1.33617, 1.7592}	17.8418	ar	4517.	7328.07	{6272., 8665.}	62.2331
48	R0	{1.24366, 4.25814}	{1.61967, 19.5409}	{{1.48217, 18.6217}, {1.82534, 20.}}	{30.234, 358.908}	inf	131.	604.135	{475.305, 766.902}	361.172	0.654982	newly	260.	231.71	{174., 296.}	19.5346	0.0934182	Reff	1.16755	1.40197	{1.29891, 1.51194}	20.0784	ar	3678.	7473.39	{6802., 8348.}	103.192
49	R0	{1.36017, 7.5415}	{1.52997, 19.5169}	{{1.48575, 18.9214}, {1.5962, 20.}}	{12.4839, 158.793}	inf	127.	739.235	{681.712, 805.893}	482.074	0.764	newly	116.	274.69	{247., 306.}	136.802	0.373183	Reff	1.22946	1.21611	{1.19309, 1.23768}	1.49042	ar	4178.	7352.97	{7060., 7661.}	75.9926
50	R0	{1.25333, 6.63578}	{1.57807, 19.6832}	{{1.48937, 19.1241}, {1.63724, 20.}}	{25.9105, 196.622}	inf	201.	815.668	{700.337, 917.304}	305.805	0.606089	newly	235.	299.57	{261., 340.}	27.5191	0.103219	Reff	1.0642	1.21816	{1.18471, 1.25062}	14.4667	ar	3401.	7667.12	{7255., 8002.}	125.437
